# Declining Guillain-Barré Syndrome after Campylobacteriosis Control, New Zealand, 1988–2010

**DOI:** 10.3201/eid1802.111126

**Published:** 2012-02

**Authors:** Michael G. Baker, Amanda Kvalsvig, Jane Zhang, Rob Lake, Ann Sears, Nick Wilson

**Affiliations:** University of Otago, Wellington, New Zealand (M.G. Baker, A. Kvalsvig, J. Zhang, A. Sears, N. Wilson);; Institute of Environmental Science and Research, Christchurch, New Zealand (R. Lake)

**Keywords:** Guillain-Barr**é** syndrome, Campylobacter infection, camplyobacteriosis, food safety, poultry, government regulations, public health surveillance, epidemiology, New Zealand

## Abstract

Food safety measures that lower incidence of campylobacteriosis might also prevent Guillain-Barré syndrome.

## Medscape CME Activity

Medscape, LLC is pleased to provide online continuing medical education (CME) for this journal article, allowing clinicians the opportunity to earn CME credit.

This activity has been planned and implemented in accordance with the Essential Areas and policies of the Accreditation Council for Continuing Medical Education through the joint sponsorship of Medscape, LLC and Emerging Infectious Diseases. Medscape, LLC is accredited by the ACCME to provide continuing medical education for physicians.

Medscape, LLC designates this Journal-based CME activity for a maximum of 1 *AMA PRA Category 1 Credit(s)*^TM^. Physicians should claim only the credit commensurate with the extent of their participation in the activity.

All other clinicians completing this activity will be issued a certificate of participation. To participate in this journal CME activity: (1) review the learning objectives and author disclosures; (2) study the education content; (3) take the post-test with a 70% minimum passing score and complete the evaluation at www.medscape.org/journal/eid; (4) view/print certificate.

**Release date: January 26, 2012; Expiration date: January 26, 2013**

## Learning Objectives

Upon completion of this activity, participants will be able to:

- Distinguish the infection most closely associated with GBS
- Analyze the temporal relationship between campylobacteriosis and GBS
- Assess differences in the association between campylobacteriosis and GBS based on age
- Evaluate the effect of infection-control measures on rates of campylobacteriosis and GBS

## CME Editor

**P. Lynne Stockton, VMD, MS, ELS(D),** Technical Writer/Editor, *Emerging Infectious Diseases. Disclosure: P. Lynne Stockton, VMD, MS, ELS(D), has disclosed no relevant financial relationships*.

## CME Author

**Charles P. Vega, MD,** Health Sciences Clinical Professor; Residency Director, Department of Family Medicine, University of California, Irvine. *Disclosure: Charles P. Vega, MD, has disclosed no relevant financial relationships.*

## Authors

***Michael G. Baker, MBChB, DPH, FNZCPHM; Amanda Kvalsvig, MBChB; Jane Zhang, MSc; Rob Lake, PhD; Ann Sears, MBChD, MPH;*** *and* ***Nick Wilson, MBChD, MPH, FNZCPHM,*** *have disclosed no relevant financial relationships.*
