# Supplementary material for: Declining Guillain-Barré Syndrome after Campylobacteriosis Control, New Zealand, 1988–2010
Source: Emerg Infect Dis. 2012 Feb;18(2):226–33. doi: 10.3201/eid1802.111126 (PMC3310455; doi:10.3201/eid1802.111126)
Supplement: Technical Appendix — Calculation of estimated repeat hospitalizations and incident cases of campylobacteriosis, New Zealand, 1988–2010. [file 11-1126-Techapp_3p.pdf]

Article DOI: http://dx.doi.org/10.3201/eid1802.111126

Declining Guillain-Barré Syndrome after Campylobacteriosis Control, New Zealand, 1988–2010

Technical Appendix

Technical Appendix Table 1. Incidence of GBS hospitalizations and adjusted estimates of hospitalization rates for incident cases by year, New Zealand, 1988–2010*

Year	Total New Zealand population	GBS total hospitalizations (principal and additional), T	GBS repeat hospitalizations in current year, Rc	GBS repeat hospitalizations from previous years, Rp	GBS repeat hospitalizations estimated from before 1988, Re (Re = C × [T – Rc])	GBS incident hospitalizations, I (I = T – Rc – Rp – Re)	GBS rate/ 100,000 (based on incident cases I)
1988	3,317,000	140	41	13	11	75	2.26
1989	3,330,200	97	29	11	6	51	1.53
1990	3,362,500	147	56	12	8	71	2.11
1991	3,495,800	147	57	16	6	68	1.95
1992	3,533,000	205	87	15	6	97	2.75
1993	3 573,600	181	84	14	3	80	2.24
1994	3,621,600	261	150	24	3	84	2.32
1995	3,675,800	194	86	21	2	85	2.31
1996	3,733,900	214	102	23	2	87	2.33
1997	3,782,600	244	110	28	–	106	2.80
1998	3,815,800	215	100	21	–	94	2.46
1999	3,837,300	210	101	28	–	81	2.11
2000	3,860,100	211	111	29	–	71	1.84
2001	3,887,000	282	148	30	–	104	2.68
2002	3,951,200	300	163	29	–	108	2.73
2003	4,027,700	295	175	26	–	94	2.33
2004	4,088,700	262	143	28	–	91	2.23
2005	4,136,000	302	149	32	–	121	2.93
2006	4,186,900	212	90	23	–	99	2.36
2007	4,230,700	223	97	27	–	99	2.34
2008	4,271,100	276	141	28	–	107	2.51
2009	4,318,100	203	96	17	–	90	2.08
2010	4,367,800	207	84	20	–	93	2.13
Total		5,028	2,400	515	47	2,056	2.32

*GBS, Guillan-Barré syndrome
Notes:
1. The populations used in this paper are from Statistics New Zealand: 1991-2010: "National population estimates, mean year ended 31 December 1991–2010 – tables", (http://www.stats.govt.nz/browse_for_stats/population/estimates_and_projections/national-pop-estimates.aspx)
1988-1990: "Estimated total population by sex, year ended 30 December (1926–2010) and 30 June (1937–2011) – tables", (http://www.stats.govt.nz/browse_for_stats/population/estimates_and_projections/historical-population-tables.aspx)
2. T = total hospitalizations with a GBS diagnostic code as the principal or additional diagnosis. These hospitalization data were provided by the New Zealand Ministry of Health.
3. Rc = repeat GBS hospitalizations during that year (i.e., same NHI number with GBS diagnostic code as the principal or additional diagnosis).
4. Rp = GBS cases with hospitalization in a previous year.
5. Re = estimated GBS repeat hospitalizations of cases first admitted before 1988, based on applying a correction factor C to cases seen in that year (T-Rc) where Re = C*(T-Rc). See the notes to Technical Appendix Table 2 for a description of how C was calculated.
6. GBS incident hospitalizations (I) where I = T-Rc-Rp-Re.

Technical Appendix Table 2. Estimating a correction factor (C) for the early years of the study by using empirical data from more recent years

Year	2000		1999		1998		1997		1996		Average C
	Rb	C	Rb	C	Rb	C	Rb	C	Rb	C	
S	29	0.290	28	0.257	21	0.183	28	0.209	23	0.205	0.229
S+1	16	0.133	15	0.174	14	0.147	5	0.051	16	0.131	0.127
S+2	12	0.100	15	0.126	14	0.165	12	0.129	2	0.021	0.108
S+3	5	0.051	10	0.085	12	0.103	11	0.134	9	0.100	0.095
S+4	5	0.052	4	0.041	7	0.061	11	0.096	11	0.134	0.077
S+5	10	0.076	5	0.052	3	0.031	5	0.044	9	0.080	0.057
S+6	0	0.000	9	0.069	5	0.052	2	0.021	5	0.044	0.037
S+7	3	0.029	0	0.000	9	0.069	5	0.052	2	0.021	0.034
S+8	1	0.009	3	0.029	0	0.000	8	0.062	3	0.032	0.027
S+9	1	0.011	1	0.009	1	0.010	0	0.000	8	0.062	0.018

Notes:
1. S = the start year for each series, e.g., 2000, for the first series; 1999 for the second series, 1998 for the third series.
2. Rb = the number of repeat hospitalizations in that year of GBS cases first admitted before the specified start year S, but with no repeat hospitalizations between year S and the current year.
3. C = the correction factor, i.e., the proportion of cases in each year with a previous hospitalization before the start year S. It is calculated using the formula C = Rb/(T-Rc). See Appendix Table 1 for values for T and Rc.
4. Averaging C over the 5 series produced a set of C values for years 0–9, which were then applied to the first 10 years of the study period (1988 to 2007).

Technical Appendix Table 3. Incidence of campylobacteriosis notifications by year, New Zealand 1988–2010

Year	Total New Zealand population	Campylobacteriosis notifications	Campylobacteriosis notification rate/100,000 population
1988	3,317,000	2,796	84.29
1989	3,330,200	4,187	125.73
1990	3,362,500	3,850	114.50
1991	3,495,800	4,148	118.66
1992	3,533,000	5,144	145.60
1993	3,573,600	8,101	226.69
1994	3,621,600	7,714	213.00
1995	3,675,800	7,442	202.46
1996	3,733,900	7,635	204.48
1997	3,782,600	8,924	235.92
1998	3,815,800	11,572	303.27
1999	3,837,300	8,161	212.68
2000	3,860,100	8,418	218.08
2001	3,887,000	10,146	261.02
2002	3,951,200	12,494	316.21
2003	4,027,700	14,788	367.16
2004	4,088,700	12,215	298.75
2005	4,136,000	13,836	334.53
2006	4,186,900	15,874	379.13
2007	4,230,700	12,778	302.03
2008	4,271,100	6,694	156.73
2009	4,318,100	7,177	166.21
2010	4,367,800	7,346	168.19

Notes:
1. Population data are estimates from Statistics New Zealand (see Table 1 footnote for details).
2. Campylobacteriosis notification data are from the Institute of Environmental Science and Research (ESR). See reference 9 in main paper.

Technical Appendix Table 4. Campylobacteriosis hospitalizations by year, New Zealand 1995–2010

Year	Total New Zealand population	Principal diagnosis	Additional diagnosis	Total, T	Repeat hospitalizations in current year, Rc	Repeat hospitalizations from previous years, Rp	Incident hospitalizations, I<br>I = T – Rc – Rp	Hospitalization rate per 100,000 population (based on incident cases (I)
1995	3,675,800	127	61	188	4	0	184	5.01
1996	3,733,900	225	150	375	7	1	367	9.83
1997	3,782,600	306	155	461	13	6	442	11.69
1998	3,815,800	363	161	524	18	4	502	13.16
1999	3,837,300	278	119	397	14	2	381	9.93
2000	3,860,100	410	101	511	16	8	487	12.62
2001	3,887,000	520	120	640	15	4	621	15.98
2002	3,951,200	586	147	733	21	5	707	17.89
2003	4,027,700	770	197	967	28	8	931	23.11
2004	4,088,700	749	174	923	35	10	878	21.47
2005	4,136,000	885	212	1,097	52	19	1026	24.81
2006	4,186,900	990	213	1,203	62	14	1127	26.92
2007	4,230,700	750	183	933	45	17	871	20.59
2008	4,271,100	395	101	496	24	10	462	10.82
2009	4,318,100	480	104	584	23	10	551	12.76
2010	4,367,800	516	101	617	19	8	590	13.51

Notes:
1. Because campylobacteriosis was recorded as a separate diagnosis from July 1995, figures for that year are incomplete.
2. Data from 2010 are provisional and may undercount hospitalizations.
