# Supplementary material for: Declining Guillain-Barré Syndrome after Campylobacteriosis Control, New Zealand, 1988–2010
Source: Emerg Infect Dis. 2012 Feb;18(2):226–33. doi: 10.3201/eid1802.111126 (PMC3310455; doi:10.3201/eid1802.111126)
Supplement: Related texts

# Research article

**DOI:** 10.3201/eid1802.111126

## Abstract

Infection with *Campylobacter* spp. commonly precedes Guillain-Barré syndrome (GBS). We therefore hypothesized that GBS incidence may have followed a marked rise and then decline in campylobacteriosis rates in New Zealand. We reviewed records for 1988–2010: hospitalization records for GBS case-patients and campylobacteriosis case-patients plus notifications of campylobacteriosis. We identified 2,056 first hospitalizations for GBS, an average rate of 2.32 hospitalizations/100,000 population/year. Annual rates of hospitalization for GBS were significantly correlated with rates of notifications of campylobacteriosis. For patients hospitalized for campylobacteriosis, risk of being hospitalized for GBS during the next month was greatly increased. Three years after successful interventions to lower *Campylobacter* spp. contamination of fresh poultry meat, notifications of campylobacteriosis had declined by 52% and hospitalizations for GBS by 13%. Therefore, regulatory measures to prevent foodborne campylobacteriosis probably have an additional health and economic benefit of preventing GBS.

Guillain-Barré syndrome (GBS) is an autoimmune condition that affects the peripheral nervous system. Patients typically describe ascending weakness and sensory disturbance that evolve over several days; during this acute phase, approximately one third of patients require ventilatory support. The condition is generally self-limiting, but for 3%–10% of patients, it is fatal (*1*).

An estimated 40%–70% of patients with GBS had an infection before GBS onset; for 6%–39% of these patients, the infection affected the gastrointestinal system (*2*). Campylobacteriosis is the most commonly identified antecedent infection; several studies have shown that in industrialized countries (Europe, North and South America, Japan, and Australia), *Campylobacter* spp*.* infection preceded GBS for 20%–50% of patients (*3**,**4*).

During 1980–2006 in New Zealand, incidence of campylobacteriosis steadily increased. The notification rate in 2006 (379 cases/100,000 population) remains the highest national rate reported in the literature (*5**,**6*). In 2006, in response to this high incidence, New Zealand introduced an array of voluntary and regulatory interventions to reduce contamination of poultry with *Campylobacter* spp (*7*). By 2008, the rate of campylobacteriosis notifications had dropped to 157 cases/100,000 population, a decrease of 59% over 2 years (*7*); this decline has persisted (*8*). Given the known association between *Campylobacter* spp. infection and GBS and the marked recent changes in reported rates of campylobacteriosis in New Zealand, we examined GBS hospitalization data for evidence of responsiveness to trends in campylobacteriosis incidence.

## Methods

### Identification of GBS Incidence

Because GBS is a serious illness that nearly always results in hospitalization, hospitalization data provided the most accurate available measure of GBS incidence. We obtained national hospital discharge data for the 23-year period 1988–2010 in New Zealand. To estimate the case-fatality proportion, we also obtained data on deaths from GBS for 1988–2008 (the most recent year available). Both datasets are collated and maintained by the New Zealand Ministry of Health.

Although hospitalization data are available for earlier years, we used 1988 as the starting point because that is when use of unique patient identifiers, the National Health Index (NHI), became universal in New Zealand. Use of the NHI enables identification and removal of repeat GBS hospitalizations for the same patient, thereby identifying the first GBS hospitalization for each case (hereafter called GBS hospitalization), which provides an estimate of the number of incident cases of GBS.

We selected all cases from 1988 on that had International Classification of Diseases, 9th and 10th Revisions, Clinical Modification and Australian Modification, codes for GBS (ICD-9 CM 357.0 and ICD-10 AM G61.0) recorded as the principal or additional diagnosis. Records of patients who had been transferred between hospitals were merged to create 1 hospitalization event. We identified repeat hospitalizations for the current year and for previous years, i.e., case-patients with the same NHI number previously admitted in the same or a previous year. Some patients were readmitted before universal use of the NHI in 1988, so the calculation needed to take these estimated repeat hospitalizations into account. (See Technical Appendix Tables 1, 2, for a description of how estimated repeat hospitalizations and incident cases were calculated.)

### Identification of Campylobacteriosis Incidence

Since 1980, campylobacteriosis has been a notifiable disease in New Zealand. Medical practitioners are required to report all identified and suspected cases to the local medical officer of health. These data are in turn collated nationally by the Institute of Environmental Science and Research for the New Zealand Ministry of Health. We used published annual totals of notifications (*9*) as well as anonymized datasets of notified cases. Most cases were culture confirmed (>96% during 1997–2008 [*7*]), although the case definition also allows for cases epidemiologically linked to a confirmed case.

Hospitalizations for campylobacteriosis are recorded in hospital discharge data, which are electronically available for a similar period. However, a specific diagnostic code for *Campylobacter* spp. infection was not introduced until July 1995. Hospitalizations for campylobacteriosis were defined as those with ICD-9 CM code 008.43 from July 1995 on and ICD-10 AM code A04.5 from July 1999 on. To create a dataset of incident cases, we included principal or additional diagnoses, merged records for those transferred with records from preceding hospitalizations, and removed repeat hospitalizations in the current and previous years.

### Analysis of Hospitalizations for GBS after Campylobacteriosis

To assess the association between the 2 conditions, we investigated the incidence of GBS among patients hospitalized for campylobacteriosis. Because campylobacteriosis was only specifically identified in hospitalization data from July 1995, this analysis focused on the period starting in July 1995. To allow a follow-up period for GBS cases to emerge, we continued the inclusion period through December 2008.

For those cases identified, we first analyzed the time from hospital admission for campylobacteriosis to admission for GBS. For epidemiologic purposes, the risk period for GBS after *Campylobacter* spp. infection is ≈2 months (*10*); neurologic signs of GBS usually develop 1–3 weeks after a preceding infection (*3*). In our dataset, a clear trend was seen toward a close temporal association between hospitalization dates: for most (34/35, 97.1%) patients, hospitalizations for GBS and campylobacteriosis were concurrent (patients were discharged with a diagnosis of both), or hospitalization for GBS occurred within 1 month of hospitalization for campylobacteriosis.

To assess the risk for GBS associated with campylobacteriosis, we calculated GBS hospitalization rates for comparison conditions, notably other infections that might be associated with an elevated risk for GBS. We used the GBS rate in the total New Zealand population as our reference rate for calculating age-standardized rate ratios for GBS after campylobacteriosis and other conditions of interest.

We also evaluated which age groups might be more vulnerable to development of GBS. To do so, we compared the age distributions of all patients hospitalized for GBS and those associated with campylobacteriosis with the age distributions for those with campylobacteriosis alone (hospitalized or with notified case).

### Statistical Analyses

Because of marked changes in campylobacteriosis disease incidence and some changes in case identification during the 23-year study period, some outcomes were measured over a shorter time. The periods associated with implementation of the *Campylobacter* spp. control interventions used a baseline period similar to that used in a previous study (*7*).

Data were analyzed by using Stata version 11.0 (StataCorp LP, College Station, TX, USA) and SAS version 9.1 (SAS Institute, Cary, NC, USA). CIs are given at the 95% level throughout. We used well-documented methods for calculating adjusted rates, rate ratios (RRs), and 95% CIs (*11*). Rates were calculated by using mean population estimates published by Statistics New Zealand (www.stats.govt.nz/browse_for_stats/population/estimates_and_projections/national-pop-estimates.aspx) as denominators. To calculate age-standardized rates, we used the population age structure determined by the New Zealand 2006 Census of Population and Dwellings (www.stats.govt.nz/Census/2006CensusHomePage/classification-counts-tables/about-people/age.aspx).

## Results

### GBS Incidence

This study identified 2,056 first hospitalizations for GBS that occurred during 1988–2010, resulting in an average rate of 2.32 hospitalizations/100,000 population/year (Technical Appendix Table 1). Incidence was not stable over the period of the study (Figure). The minimum recorded rate was 1.53 hospitalizations/100,000 population/year in 1989; the maximum was 2.93 in 2005. During 1989–2008, a total of 56 deaths from GBS were recorded; case-fatality proportion (56 deaths/1,873 cases) was 3.0%.

**Figure:** Guillain-Barré syndrome (GBS) hospitalization rates and campylobacteriosis notification rates, by year, New Zealand, 1988–2010. *Per 100,000 population.

### Changes in GBS and Campylobacteriosis Incidence

For 1988–2010, there was a significant direct correlation between annual rates of hospitalization for GBS and annual rates of notification of campylobacteriosis cases (Spearman ρ = 0.52, p = 0.012). During 1988–2006, incidence of campylobacteriosis notifications and of GBS hospitalizations increased (Figure; Technical Appendix Table 3). Subsequently, campylobacteriosis notifications then decreased markedly, and GBS hospitalizations decreased, although less dramatically. The fall in campylobacteriosis notifications followed the introduction of countrywide campylobacteriosis control measures focused on reducing contamination levels in fresh poultry meat (*7*).

Table 1 summarizes the changes between the 2 periods: 1) 2002–2006, the baseline period, when rising campylobacteriosis rates became an urgent public health concern, and 2) 2008–2010, the postintervention period, after implementation of wide-ranging control measures. The transition year, 2007, was excluded.

**Table 1:** Incidence of campylobacteriosis and GBS before and after intervention to reduce *Campylobacter* spp. in poultry, New Zealand 2002–2010*

Incident condition	Before intervention, 2002–2006				After intervention, 2008–2010†				Change	
	Total no.	Average/year	Rate‡		Total no.	Average/year	Rate‡		Rate ratio (95% CI)	p value
Campylobacteriosis notifications§	69,207	13,841	339.4		21,217	7,072	163.8		0.48 (0.48–0.49)	<0.0001
Campylobacteriosis hospitalizations¶	4,669	934	23.2		1,603	534	12.2		0.53 (0.51–0.54)	<0.0001
GBS hospitalizations¶	513	103	2.6		290	97	2.2		0.87 (0.81–0.93)	0.0496

*GBS, Guillain-Barré syndrome. †Excludes 2007, which was a transitional year. ‡Annual no. cases/100,000 person-years at risk. Denominator populations based on mean population estimates published by Statistics New Zealand (www.stats.govt.nz/browse_for_stats/population/estimates_and_projections/national-pop-estimates.aspx). Campylobacteriosis and Guillan-Barré syndrome hospitalizations used age-standardized rates based on the age structure of the New Zealand 2006 Census of Population and Dwellings (www.stats.govt.nz/Census/2006CensusHomePage/classification-counts-tables/about-people/age.aspx). §Published campylobacteriosis notification data (*9*). ¶Hospitalization data from New Zealand Ministry of Health.

During the postintervention period, notifications and hospitalizations decreased by ≈50% (Technical Appendix Tables 3, 4). Incidence of GBS declined by 13%, which was statistically significant (RR 0.87, 95% CI 0.81–0.93), suggesting that ≈25% of GBS was caused by preceding campylobacteriosis.

### GBS among Patients Hospitalized for Campylobacteriosis or Other Conditions

During 1995–2008, among the 8,448 patients hospitalized for campylobacteriosis, 35 were also hospitalized for GBS. The frequency distribution of time delays is shown in Table 2. These data show that most (29) of these 35 patients had diagnoses of GBS and campylobacteriosis at time of hospital discharge. Another 5 patients were hospitalized for GBS within 4 weeks of being hospitalized for campylobacteriosis. The time difference for the remaining patient was >1,500 days (this patient was excluded from subsequent analyses). This striking distribution further supports a causative association between campylobacteriosis and GBS in New Zealand.

**Table 2:** Hospitalization for Guillain-Barré syndrome during or after hospitalization for campylobacteriosis, New Zealand, July 1995–December 2008

Interval, d	No. (%) persons hospitalized	Cumulative %
Concurrent	29 (82.9)	82.9
1–7	2 (5.7)	88.6
8–28	3 (8.6)	97.1
1,524 (4.2 y)	1 (2.9)	100
Total	35 (100)	Not applicable

We calculated the rate of GBS hospitalizations among the cohort of patients hospitalized for campylobacteriosis and compared this with rates of GBS hospitalization among other patient cohorts hospitalized for infectious diseases (Table 3). This analysis used the overall rate of GBS hospitalizations among the New Zealand population as a reference for calculating age-standardized RRs.

**Table 3:** Incidence of GBS after hospitalization for campylobacteriosis and other infectious diseases compared with total population incidence rate for GBS, New Zealand, July 1995–December 2008*

Initial hospitalization condition	ICD-9 codes	ICD-10 codes	Denominator population†	Subsequent GBS hospitalizations (concurrent hospitalizations)‡	Crude rate§	Age-standardized rate¶ (95% CI)	Age-standardized rate ratio (95% CI)
Infectious diseases (ICD chapter 1)	001–139	A00–B99	732,254	56 (273)	90.7	87.0 (56.9–116.4)	34.3 (29.2–40.3)
Pneumonia and influenza	480–488	J09–J18	250,399	19 (82)	91.1	96.2 (25.1–167.3)	37.9 (26.5–54.3)
Enteric diseases#	001–002 004–008.42 008.44–009.3	A00–A01 A03–A04.4 A04.6–A09	77,793	6 (21)	93.3	132.0 (1.2–262.7)	52.0 (32.2–84.2
Campylobacteriosis	008.43	A04.5	8,448	5 (29)	710.2	810.0 (41.4–1,578.7)	319.4 (201.5–506.4)
Salmonellosis	003	A02	2,148	0 (0)	0	0	0
New Zealand population GBS rate	NA	NA	53,617,400	1,320	2.5	2.5 (2.4–2.7)	Referent

*GBS, Guillain-Barré syndrome; ICD, International Classification of Diseases; ICD-9, ICD 9th Revision; ICD-10, ICD 10th Revision; NA, not applicable. †Denominator population based on either 1) incident hospitalizations for specific condition (number of acute and arranged first overnight hospitalizations as principal or additional diagnosis); or 2) total New Zealand population person-years for July 1995– December 2008 for calculating the New Zealand population GBS rate. ‡First hospitalization of GBS either 1) among those with a previous hospitalization in the preceding 30 d and excluding those with concurrent diagnoses (numbers in parentheses); or 2) in the total New Zealand population for July 1995–December 2008. §Rate per 100,000 person-years at risk. For GBS hospitalizations after specific conditions, monthly rate has been multiplied by 12 to convert to annual rate. ¶Standard population is population of New Zealand according to the New Zealand 2006 Census of Population and Dwellings (www.stats.govt.nz/Census/2006CensusHomePage/classification-counts-tables/about-people/age.aspx). #Excluding campylobacteriosis and salmonellosis.

The age-standardized rate of GBS was 810.0 hospitalizations/100,000 person-years (95% CI 41.4–1,578.7) in the month after hospitalization for campylobacteriosis. The RR, compared with the rate of GBS hospitalizations among the New Zealand population, was 319.4 (95% CI 201.5–506.4). This rate was markedly higher than rates for the other patient cohorts examined (Table 3).

Patients with GBS (median age 52.5 years) were significantly older than those hospitalized for campylobacteriosis (median 41 years), who in turn were significantly older than those with campylobacteriosis notifications (median 31 years) (Table 4, Table 5). The age of the subpopulation of patients with GBS associated with campylobacteriosis was similar (median 54 years) to that of the total population with GBS.

**Table 4:** Distribution of campylobacteriosis and GBS cases, by age, New Zealand, July 1995–December 2008*

Age group, y	Campylobacteriosis notifications			Camplyobacteriosis hospitalizations			GBS hospitalizations			GBS hospitalizations associated with camplylobacteriosis	
	No. (%)	Rate‡		No. (%)	Rate‡		No. (%)	Rate‡		No. (%)	Rate‡
<5	15,232 (11.7)	442.5		538 (6.4)	13.9		45 (3.4)	1.2		0	0
5–9	6,295 (4.9)	176.9		200 (2.4)	5.0		33 (2.5)	0.8		2 (5.9)	0.1
10–19	14,481 (11.2)	203.7		965 (11.4)	12.2		113 (8.6)	1.4		3 (8.8)	0
20–29	25,063 (19.3)	385.9		1,509 (17.9)	20.6		115 (8.7)	1.6		1 (2.9)	0
30–39	19,511 (15.0)	270.7		935 (11.1)	11.5		146 (11.1)	1.8		3 (8.8)	0
40–49	16,572 (12.8)	237.0		747 (8.8)	9.6		149 (11.3)	1.9		5 (14.7)	0.1
50–59	14,311 (11.0)	261.9		778 (9.2)	13.0		226 (17.1)	3.8		6 (17.7)	0.1
60–69	9,559 (7.4)	255.7		824 (9.8)	19.9		209 (15.8)	5.0		7 (20.6)	0.2
70–79	6,174 (4.8)	235.9		1,046 (12.4)	35.9		200 (15.2)	6.9		5 (14.7)	0.2
>80	2,712 (2.1)	190.8		906 (10.7)	57.9		84 (6.4)	5.4		2 (5.9)	0.1
Total	129,910 (100.0)	270.4		8,448 (100.0)	15.8		1,320 (100.0)	2.5		34 (100.0)	0.1

*GBS, Guillain-Barré syndrome. †Association with hospitalization for camplylobacteriosis. Includes subsequent and concurrent hospitalizations (campylobacteriosis and GBS diagnoses at time of hospital discharge). ‡Average annual no./100,000 population.

**Table 5:** Comparsion of ages of patients with campylobacteriosis and GBS, New Zealand, July 1995–December 2008*

Calculation	A. Patients with campylobacteriosis		B. Patients hospitalized for campylobacteriosis			C. Patients hospitalized for GBS			D. Patients hospitalized for GBS and campylobacteriosis‡	
	Age, y		Age, y	p value for age compared with A§		Age, y	p value for age compared with B§		Age, y	p value for age compared with C§
Mean	33.7		43.7	<0.0001		48.8	<0.0001		50.3	0.7063
Median	31		41	<0.0001		52.5	<0.0001		54	0.7280

*GBS, Guillain-Barré syndrome. †Referent. ‡Includes subsequent and concurrent hospitalizations (campylobacteriosis and GBS diagnoses at time of hospital discharge). §Means compared with Student *t*-test medians compared with median 2-sample test.

## Discussion

This study shows how the incidence of an acute infectious disease, campylobacteriosis, can influence incidence of a serious neurologic condition, GBS. At the population level, hospitalizations for GBS were significantly correlated with notifications of campylobacteriosis for the same year. At the individual level, compared with rates for the New Zealand population as a whole, hospitalizations for campylobacteriosis were associated with an almost 320-fold increased risk for subsequent hospital admission for GBS in the next month.

Results also show that food safety measures to reduce contamination of fresh poultry meat with *Campylobacter* spp. not only reduced incidence of campylobacteriosis but also were associated with reduced incidence of GBS. In the 3 years after introduction of these control measures, campylobacteriosis notifications and hospitalizations decreased by ≈50%, and GBS hospitalizations dropped by 13%. These findings suggest that in New Zealand, *Campylobacter* spp. infection may be responsible for ≈25% of GBS cases, which is consistent with data from other industrialized countries (*3*).

A recent systematic review (*12*) summarized attempts to quantify the association between campylobacteriosis and GBS incidence. There is general agreement that measuring GBS population rates is useful, for example, for monitoring vaccine adverse effects (*13**,**14*). However, to our knowledge, no similar population-based analysis of the relationship between GBS and campylobacteriosis has been conducted for other countries, probably because few countries collect similarly detailed national-level hospitalization data. An earlier population-based study in New Zealand did not show an association between notifications for campylobacteriosis and GBS incidence (*15*). However, that study was over a shorter period and did not use a correction factor to account for undetected repeat hospitalizations in the early years of the observation period, which would have made it harder to detect an association between incidence rates for the 2 conditions.

Compared with global estimates, rates of GBS in New Zealand are high. In a review of reported GBS rates during 1980–2000, worldwide incidence varied between 1.0 and 1.8 cases/100,000 population/year (*2*). The average reported rate for New Zealand during this period was at the upper end of this range (1.8/100,000). A more recent study from the United States estimated that annual hospitalization rates for GBS varied between 1.65 and 1.79/100,000 during 2000–2004 (*16*). In New Zealand during the same period, the annual hospitalization rates varied between 1.8 and 2.7/100,000.

The 320-fold increased risk for GBS in the month after hospitalization for campylobacteriosis found in this study is higher than that previously reported. In a case–control study of GBS and potential antecedent infections in the United Kingdom, Tam et al. reported that persons with *Campylobacter* enteritis had a 38-fold increased risk that GBS would develop in the next 2 months (*17*). However, when they added a correction factor to account for under-ascertainment of campylobacteriosis, the risk increased to 60-fold. Similarly, a population-based study in Sweden estimated that patients with laboratory-confirmed *C. jejuni* infection had a 100-fold increased risk that GBS would develop in the next 2 months (*10*). We used a 1-month risk period because the GBS cases we identified subsequent to hospitalizations for campylobacteriosis were confined to this period. Using a 2-month risk period would have halved our estimated age-standardized RR, but the elevated risk would still be higher than that reported elsewhere.

The proportion of GBS cases attributable to preceding *Campylobacter* spp. infection estimated for New Zealand (≈25%) is within the range described elsewhere. Studies from other countries and regions have reported serologic evidence of previous *C. jejuni* infection in 13%–72% of GBS case-patients (*18*). A systematic review, based on 32 eligible studies, estimated that 31% of GBS cases were attributable to *Campylobacter* spp. infection (*12*). The strength of the association with GBS may vary geographically, according to the neuropathic propensity of local *Campylobacter* strains. We would also expect the percentage contribution of preceding *Campylobacter* spp. infection to vary according to the incidence of this infection in the population and the incidence of other causal infections and exposures.

The results of our study suggest that risk for GBS may not be uniform for different degrees of campylobacteriosis severity. Our study found that risk for GBS was ≈1 in 1,690 (5 in 8,448) among patients hospitalized for campylobacteriosis and that ≈25% of GBS cases were caused by campylobacteriosis. On the basis of an annual incidence of ≈100 GBS cases, these data suggest that ≈42,000 cases of campylobacteriosis occur each year in New Zealand. Current estimates of total campylobacteriosis incidence are higher. Annual notifications remain at ≈7,000 cases. A study from the United Kingdom estimated that 9.3 cases of campylobacteriosis occurred in the community for every notified case (*19*); a study from Australia estimated this number to be 10 (*20*). Applied to New Zealand, these multipliers suggest an incidence among the population of 65,000 to 70,000 cases per year. These findings suggest that the causal association between campylobacteriosis and GBS is probably weaker for patients with less severe infections, who do not require hospitalization.

Analysis of the age distribution of patients with campylobacteriosis and GBS suggests that older age is a major risk factor for more severe outcomes (hospitalization and GBS) from this enteric infection. The rising incidence of GBS with increasing age in New Zealand is consistent with incidence observed in other countries (*21*).

One strength of this study is that it has been able to monitor a natural experiment in which campylobacteriosis incidence decreased by 50% within a few months, providing an unusual opportunity to assess the effect of this change on incidence of GBS. New Zealand’s comprehensive recording of national hospitalization data and use of a unique patient number also provided us with a consistent base for estimating population rates of GBS over a prolonged period. Although the spectrum of GBS includes extremely mild cases, studies elsewhere indicate that only ≈3.0%–5.8% of patients with GBS are not hospitalized (*22**,**23*). In addition, patients with *Campylobacte*r-associated GBS are believed to experience more severe disease (*24**,**25*), which would minimize the number of *Campylobacter*-associated GBS cases missed by this investigation.

One limitation of this study is the group used to compare risk for GBS: the total New Zealand population. A variety of conditions and events have been identified as possible GBS triggers (*1**,**24**,**26**–**29*). Consequently, because it is not possible with current knowledge to identify a reference patient population with no additional GBS risk factors, we considered that the total population provided the most appropriate reference rate.

The association between campylobacteriosis and GBS in New Zealand needs further investigation. It will be useful to continue to follow the trends identified here to assess the stability of the decrease in GBS, which will eventually give greater precision to the estimated contribution of campylobacteriosis. Ongoing monitoring of GBS should be included in the comprehensive surveillance of infectious diseases (*30*). The hypothesis that patients not hospitalized for campylobacteriosis have a lower risk for GBS should be tested by investigation of incidence of GBS among these patients.

Our findings suggest the value of further research to identify other potentially preventable infectious causes of GBS. Table 3 shows a markedly elevated risk for GBS after hospitalization for infectious diseases in general. Investigating these associations in detail may identify other potentially preventable causes of GBS.

Findings of this study have relevant implications for food safety programs. Although GBS is rare, the toll it takes on the individual patient is often high (*1*). Even with treatment, 9%–17% of patients die or remain disabled (*31*), and repeat hospitalizations are common, representing ≈60% of total hospitalizations (Technical Appendix Table 1). Almost half of all patients report ongoing difficulties 3–6 years after GBS onset (*32*). Consequently, ongoing health care costs for each GBS patient are considerable. In New Zealand during 1988–2008, the GBS case-fatality proportion was 3.0%, and a recent article (*33*) estimated that 204 (13%) of 1,568 disability-adjusted life years for campylobacteriosis in New Zealand were caused by GBS.

This study shows that food safety programs that successfully lower rates of campylobacteriosis might have the additional benefit of preventing GBS. This finding adds to the health and economic arguments for such control measures. The justification for such interventions is particularly strong where a substantial proportion of human disease can be linked to a widely consumed food source, such as contaminated poultry products, as it is in New Zealand (*7*).

## Supplementary Material

Technical Appendix Calculation of estimated repeat hospitalizations and incident cases of campylobacteriosis, New Zealand, 1988–2010.

## References

[1] van Doorn PA, Ruts L, Jacobs BC. Clinical features, pathogenesis, and treatment of Guillain-Barré syndrome. Lancet Neurol. 2008;7:939–50. 10.1016/S1474-4422(08)70215-118848313

[2] McGrogan A, Madle GC, Seaman HE, de Vries CS. The epidemiology of Guillain-Barré syndrome worldwide. Neuroepidemiology. 2009;32:150–63. 10.1159/00018474819088488

[3] Jacobs BC, van Belkum A, Endtz HP. Guillain-Barré syndrome and *Campylobacter* infection. In: Nachamkin I, Szymanski CM, Blaser MJ, editors. *Campylobacter*, 3rd ed. Washington: ASM Press; 2008. p. 245–62.

[4] Nachamkin I, Allos BM, Ho T. *Campylobacter* species and Guillain-Barré syndrome. Clin Microbiol Rev. 1998;11:555–67.966598310.1128/cmr.11.3.555PMC88896

[5] Baker M, Wilson N, Ikram R, Chambers S, Shoemack P, Cook G. Regulation of chicken contamination is urgently needed to control New Zealand's serious campylobacteriosis epidemic. N Z Med J. 2006;119:U2264.17063200

[6] Baker MG, Sneyd E, Wilson N. Is the major increase in notified campylobacteriosis in New Zealand real? Epidemiol Infect. 2007;135:163–70. 10.1017/S095026880600658316753076PMC2870549

[7] Sears A, Baker MG, Wilson N, Marshall J, Muellner P, Campbell DM, Marked campylobacteriosis decline after interventions aimed at poultry, New Zealand. Emerg Infect Dis. 2011;17:1007–15. 10.3201/eid1706.10127221749761PMC3358198

[8] Baker MG, Sears A, Wilson N, French N, Marshall J, Muellner P, Keep the focus on contaminated poultry to further curtail New Zealand’s campylobacteriosis epidemic. N Z Med J. 2011;124:135–9.21946979

[9] Institute of Environmental Science and Research Ltd. Notifiable and other diseases in New Zealand: annual report 2010. Porirua (New Zealand): The Institute; 2011.

[10] McCarthy N, Giesecke J. Incidence of Guillain-Barré syndrome following infection with *Campylobacter jejuni.* Am J Epidemiol. 2001;153:610–4. 10.1093/aje/153.6.61011257070

[11] Rothman K, Greenland S, Lash T, eds. Modern epidemiology, 3rd ed. Philadelphia: Lippincott Williams & Wilkins; 2008.

[12] Poropatich KO, Walker CL, Black RE. Quantifying the association between *Campylobacter* infection and Guillain-Barré syndrome: a systematic review. J Health Popul Nutr. 2010;28:545–52. 10.3329/jhpn.v28i6.660221261199PMC2995022

[13] Black S, Eskola J, Siegrist C, Halsey N, MacDonald N, Law B, Importance of background rates of disease in assessment of vaccine safety during mass immunisation with pandemic H1N1 influenza vaccines. Lancet. 2009;374:2115–22. 10.1016/S0140-6736(09)61877-819880172PMC2861912

[14] DeStefano F, Tokars J. H1N1 vaccine safety monitoring: beyond background rates. Lancet. 2010;375:1146–7. 10.1016/S0140-6736(09)61917-619880171

[15] Lake R, Baker M, Nichol C, Garrett N. Lack of association between long-term illness and infectious intestinal disease in New Zealand. N Z Med J. 2004;117:U893.15156211

[16] Alshekhlee A, Hussain Z, Sultan B, Katirji B. Guillain-Barré syndrome. Neurology. 2008;70:1608–13. 10.1212/01.wnl.0000310983.38724.d418443311

[17] Tam CC, O’Brien SJ, Petersen I, Islam A, Hayward A, Rodrigues LC. Guillain-Barré syndrome and preceding infection with *Campylobacter*, influenza and Epstein-Barr virus in the general practice research database. PLoS ONE. 2007;2:e344. 10.1371/journal.pone.000034417406668PMC1828628

[18] Hadden RD, Gregson NA. Guillain-Barré syndrome and *Campylobacter jejuni* infection. Symp Ser Soc Appl Microbiol. 2001;30:145S–54S.1142257010.1046/j.1365-2672.2001.01363.x

[19] Tam CC, Rodrigues LC, Viviani L, Dodds JP, Evans MR, Hunter PR, Longitudinal study of infectious intestinal disease in the UK (IID2 study): incidence in the community and presenting to general practice. Gut. 2011 Jun 27; [Epub ahead of print]. 2170882210.1136/gut.2011.238386PMC3230829

[20] Hall G, Yohannes K, Raupach J, Becker N, Kirk M. Estimating community incidence of *Salmonella, Campylobacter*, and Shiga toxin–producing *Escherichia coli* infections, Australia. Emerg Infect Dis. 2008;14:1601–9.1882682510.3201/eid1410.071042PMC2609882

[21] Sejvar JJ, Baughman AL, Wise M, Morgan OW. Population incidence of Guillain-Barré syndrome: a systematic review and meta-analysis. Neuroepidemiology. 2011;36:123–33. 10.1159/00032471021422765PMC5703046

[22] Chiò A, Cocito D, Leone M, Giordana MT, Mora G, Mutani R. Guillain-Barré syndrome: a prospective, population-based incidence and outcome survey. Neurology. 2003;60:1146–50.1268232210.1212/01.wnl.0000055091.96905.d0

[23] Cheng Q, Jiang GX, Fredrikson S, Link H, De Pedro-Cuesta J. Incidence of Guillain-Barré syndrome in Sweden 1996. Eur J Neurol. 2000;7:11–6.10809910

[24] Hadden RDM, Karch H, Hartung H-P, Zielasek J, Weissbrich B, Schubert J, Preceding infections, immune factors, and outcome in Guillain-Barré syndrome. Neurology. 2001;56:758–65.1127431110.1212/wnl.56.6.758

[25] Van Koningsveld R, Van Doorn PA, Schmitz PIM, Ang CW, Van der Meche FGA. Mild forms of Guillain-Barré syndrome in an epidemiologic survey in the Netherlands. Neurology. 2000;54:620.1068079310.1212/wnl.54.3.620

[26] Lehmann HC, Hartung H-P, Kieseier BC, Hughes RAC. Guillain-Barré syndrome after exposure to influenza virus. Lancet Infect Dis. 2010;10:643–51. 10.1016/S1473-3099(10)70140-720797646

[27] Abbi KK, Rizvi SM, Sivik J, Thyagarajan S, Loughran T, Drabick JJ. Guillain-Barré syndrome after use of alemtuzumab (Campath) in a patient with T-cell prolymphocytic leukemia: a case report and review of the literature. Leuk Res. 2010;34:e154–6. 10.1016/j.leukres.2010.02.03620362332

[28] Kelly JJ, Karcher DS. Lymphoma and peripheral neuropathy: a clinical review. Muscle Nerve. 2005;31:301–13. 10.1002/mus.2016315543550

[29] Zhang L, Arrington S, Keung YK. Guillain-Barré syndrome after transplantation. Leuk Lymphoma. 2008;49:291–7. 10.1080/1042819070176000318231916

[30] Baker MG, Easther S, Wilson N. A surveillance sector review applied to infectious diseases at a country level. BMC Public Health. 2010;10:332. 10.1186/1471-2458-10-33220540772PMC3224743

[31] Hughes RA, Swan AV, Raphael JC, Annane D, van Koningsveld R, van Doorn PA. Immunotherapy for Guillain-Barré syndrome: a systematic review. Brain. 2007;130:2245–57. 10.1093/brain/awm00417337484

[32] Bernsen RA, de Jager AE, Schmitz PI, van der Meche FG. Long-term impact on work and private life after Guillain-Barré syndrome. J Neurol Sci. 2002;201:13–7. 10.1016/S0022-510X(02)00158-212163188

[33] Lake RJ, Cressey PJ, Campbell DM, Oakley E. Risk ranking for foodborne microbial hazards in New Zealand: burden of disease estimates. Risk Anal. 2010;30:743–52. 10.1111/j.1539-6924.2009.01269.x19645753

# Research article

**DOI:** 10.3201/eid1802.111126

## Earning CME Credit

To obtain credit, you should first read the journal article. After reading the article, you should be able to answer the following, related, multiple-choice questions. To complete the questions (with a minimum 70% passing score) and earn continuing medical education (CME) credit, please go to www.medscape.org/journal/eid. Credit cannot be obtained for tests completed on paper, although you may use the worksheet below to keep a record of your answers. You must be a registered user on Medscape.org. If you are not registered on Medscape.org, please click on the New Users: Free Registration link on the left hand side of the website to register. Only one answer is correct for each question. Once you successfully answer all post-test questions you will be able to view and/or print your certificate. For questions regarding the content of this activity, contact the accredited provider, CME@medscape.net. For technical assistance, contact CME@webmd.net. American Medical Association’s Physician’s Recognition Award (AMA PRA) credits are accepted in the US as evidence of participation in CME activities. For further information on this award, please refer to http://www.ama-assn.org/ama/pub/category/2922.html. The AMA has determined that physicians not licensed in the US who participate in this CME activity are eligible for *AMA PRA Category 1 Credits*™. Through agreements that the AMA has made with agencies in some countries, AMA PRA credit may be acceptable as evidence of participation in CME activities. If you are not licensed in the US, please complete the questions online, print the certificate and present it to your national medical association for review.

## Article Title: Guillain-Barré Syndrome and Campylobacteriosis

## CME Questions

**1. Which of the following infections is most common prior to the development of Guillain-Barré syndrome (GBS)?**A. *Listeria monocytogenes*B. *Campylobacter jejuni*C. *Neisseria meningitidis*D. *Neisseria gonorrhoeae***2. Which of the following statements best describes the temporal relationship between campylobacteriosis and GBS in the current study?**A. Most patients had concurrent campylobacteriosis and GBSB. GBS followed infection with *C. jejuni* by an average of 2 monthsC. GBS followed infection with *C. jejuni* by an average of 6 monthsD. There was no temporal relationship between campylobacteriosis and GBS**3. Which of the following age groups in the current study featured the strongest association between campylobacteriosis and GBS?**A. <5 years oldB. 5–9 years oldC. 10–19 years oldD. 60–69 years old**4. How did new food safety measures affect risks for campylobacteriosis and GBS in the current study?**A. Food safety measures failed to significantly alter the prevalence of campylobacteriosis or GBSB. Food safety measures reduced the prevalence of campylobacteriosis onlyC. Food safety measures reduced the prevalence of GBS onlyD. Food safety measures reduced the prevalence of both campylobacteriosis and GBS

## Activity Evaluation

**1:** The activity supported the learning objectives.

Strongly Disagree				Strongly Agree
1	2	3	4	5
**2. The material was organized clearly for learning to occur.**				
Strongly Disagree				Strongly Agree
1	2	3	4	5
**3. The content learned from this activity will impact my practice.**				
Strongly Disagree				Strongly Agree
1	2	3	4	5
**4. The activity was presented objectively and free of commercial bias.**				
Strongly Disagree				Strongly Agree
1	2	3	4	5
